# Eye Movement Analysis: A Kernel Density Estimation Approach for Saccade Direction and Amplitude

**DOI:** 10.3390/jemr19010010

**Published:** 2026-01-19

**Authors:** Paula Fehlinger, Bernhard Ertl, Bianca Watzka

**Affiliations:** 1 Didactics of Physics and Technology, I. Institute of Physics IA, RWTH Aachen University, Sommerfeldstraße 16, 52074 Aachen, Germany; fehlinger@physik.rwth-aachen.de; 2 Department of Human Sciences, Institute of Educational Sciences, University of the Bundeswehr Munich, Werner-Heisenberg-Weg 39, 85577 Neubiberg, Germany; bernhard.ertl@unibw.de

**Keywords:** eye tracking, amplitude and direction of saccades, solution strategies

## Abstract

Eye movements are important indicators of problem-solving or solution strategies and are recorded using eye-tracking technologies. As they reveal how viewers interact with presented information during task processing, their analysis is crucial for educational research. Traditional methods for analyzing saccades, such as histograms or polar diagrams, are limited in capturing patterns in direction and amplitude. To address this, we propose a kernel density estimation approach that explicitly accounts for the data structure: for the circular distribution of saccade direction, we use the von Mises kernel, and for saccade amplitude, a Gaussian kernel. This yields continuous probability distributions that not only improve accuracy of representations but also model the underlying distribution of eye movements. This method enables the identification of strategies used during task processing and reveals the connections to the underlying cognitive processes. It allows for a deeper understanding of information processing during learning. By applying our new method to an empirical dataset, we uncovered differences in solution strategies that conventional techniques could not reveal. The insights gained can contribute to the development of more effective teaching methods, better tailored to the individual needs of learners, thereby enhancing their academic success.

## 1. Introduction

Eye movements are the observable outcome of perceptual and attentional processes, providing insights into how learners engage with visual information [1]. Researchers capture and record these eye movements using eye tracking technologies, making procedural aspects of information processing—such as search patterns or attention allocation—visible [2]. In educational research, eye movement analysis has become increasingly important (e.g., [3,4]).

Saccade analysis provides a particularly good understanding of visual attention and cognitive processes [5]. Considering both saccade direction and amplitude makes it possible, for example, to identify and characterize learners’ processing strategies (e.g., [6]). Numerous studies have already analyzed and presented saccade metrics [7,8,9,10]. Different methods have been used to analyze saccade directions, including absolute frequencies [8,9], relative frequencies [4,10], and kernel density estimation (KDE) with a Gaussian kernel [3,7]. Many of these have visualized saccade direction using polar diagrams, particularly because they offer a clear and intuitive representation of circular data [11].

However, existing methods of saccade analysis are limited in their ability to capture the distribution of directional and amplitude data, which may result in a loss of important information. Existing studies have often treated continuous values of saccadic metrics discretely, representing them in intervals. As a result, information about individual data points is lost [12]. This discrete treatment of continuous values could mean that subtle nuances or variations in eye movements are not fully captured, which could affect the accuracy of the analysis. As an alternative, other studies often connect the discrete data points of saccades to achieve an apparently continuous representation. However, this method risks biasing the data, as the connecting lines between the discrete points may not accurately reflect the actual eye movements [13].

Although kernel density estimation for circular data has a long tradition in statistics (e.g., [11]), these methods have not yet been systematically applied to saccade directions. The contribution of the present work is to transfer this well-established mathematical framework to the analysis of saccadic movements. Moreover, directional data (saccade directions) alone and the combined analysis of direction and amplitude pose fundamentally different statistical challenges: while saccade directions follow a circular distribution, saccade amplitudes are linearly distributed. Consequently, their joint modeling requires the integration of two KDE approaches—a circular von Mises kernel for saccade directions and a Gaussian kernel for saccade amplitudes. This combination avoids artificial continuity and captures circular (directional) and linear (amplitude) distributions more faithfully than conventional techniques. Beyond providing more accurate representations, this probabilistic modeling enables predictions about likely gaze shifts and reveals distinct patterns of saccadic behavior.

## 2. Theory

### 2.1. Saccades: A Window into Cognitive Processing During Learning

**Figure 1:**
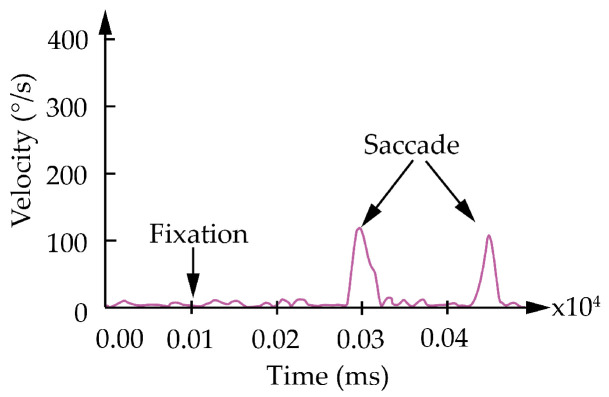
Excerpt of velocity ranges of saccades and fixations.

**Figure 2:**
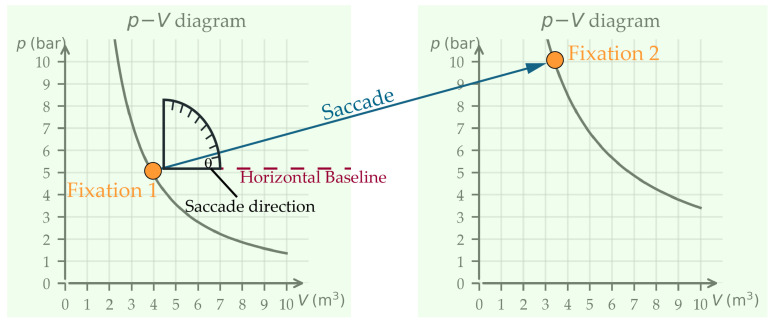
Saccade between two fixations when comparing two graphs. The saccade direction is measured absolutely with respect to the horizontal axis.

**Figure 3:**
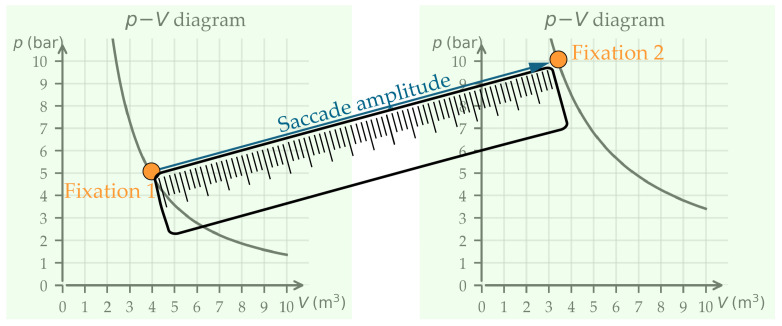
Saccade between two fixations when comparing two graphs. The saccade amplitude corresponds to the straight line between the two fixations.

Saccades are rapid and voluntary or involuntary eye movements that transit the focus of the gaze from one fixation to another [14]. Saccades have a characteristic temporal profile (see Figure 1). The eye is initially stable (start point fixation 1) and then rapidly accelerates to a peak velocity, followed by a rapid deceleration and then a rapid return to stability (end point fixation 2) (see Figure 2 and Figure 3). All of this is achieved in a relatively short time frame of 30 ms and 80 ms [14,15]. Saccades can be described either by their velocity, which is understood vectorially (i.e., with a direction and magnitude), or by their duration, direction, and amplitude [15]. For this contribution, the description of saccade direction and saccade amplitude is central. Both parameters play a crucial role in interpreting cognitive processes. Saccades are analyzed within defined Areas of Interest (AOIs). AOIs refer to clearly delineated regions of the image or task that the eye-tracking analysis specifically focuses on. These AOIs are highlighted in color, as shown in Figure 2 and Figure 3.

Saccade directions are given as angles between the saccades and the horizontal axis (see Figure 2). They reflect reading behavior of the presentation. For example, texts present information sequentially [16]. Accordingly, reading texts leads to many horizontal saccades, predominantly in the direction of reading, and (depending on reading skills) also backwards [17]. Mathematical graphs, on the other hand, are pictorial representations. Images present all information at once (see [16]). Here, saccade directions can reflect the strategy underlying the interpretation [6].

Saccade amplitudes, also known as saccade length, describe the distance between a fixation and the immediately preceding one ([5], see Figure 3). Without access to additional information, it is more difficult to interpret saccade amplitude than saccade directions. For example, when reading texts, long saccades suggest advanced readers [18]. However, when viewing pictorial representations, long saccades often signify search processes that are task- or goal-driven [19].

As the example studies demonstrate, saccade directions and amplitude offer observable features that can reflect aspects of learners’ processing strategies. However, unlike the simplified representations in Figure 2 and Figure 3, real task performance involves numerous saccades per participant. To meaningfully analyze this large number of saccades, appropriate statistical methods are required.

### 2.2. Existing Methods for Saccade Analysis and Visualization

**Figure 4:**
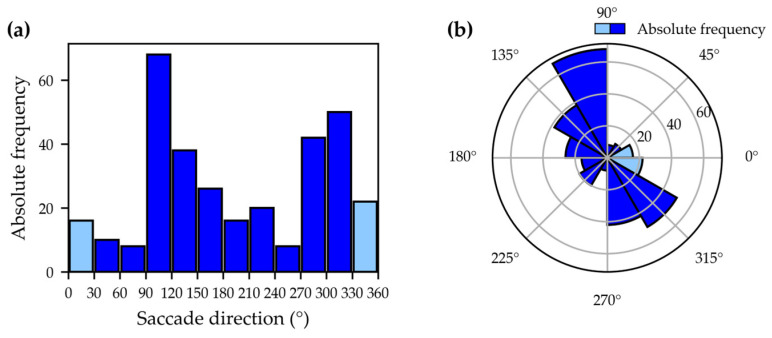
Visualization of absolute frequencies of saccade directions as a bar chart in Cartesian coordinates (**a**) and as a polar diagram (**b**). In the Cartesian plot, peaks around 0–30° and 330–360° appear to represent opposite directions. The polar representation, however, makes it clear that these directions are in close proximity, thus more accurately reflecting the circular nature of the data.

Studies from different disciplines have examined saccade visualization and its potential for the analysis of gaze strategies, using a variety of methods and graphical visualizations. In the following, we provide an overview of selected approaches and discuss their limitations. To illustrate the different visualization approaches discussed in the following sections, all example figures (Figure 4 and Figures 6–11) are based on a single exemplar eye-tracking dataset (Dataset S2, see Supplementary Materials).

#### 2.2.1. Polar Diagrams

Circular data is usually measured in radians or degrees. Due to its periodic nature, it is fundamentally different from linear data. Polar diagrams are a display method that is particularly useful for visualizing circular or periodic data. The plot employs a 360-degree scale to encompass the entire circle, illustrating all directions. The circle’s periodic nature enables a seamless representation of the data without generating discrete segments or breaks [11]. In addition to visualizing circular or periodic data such as saccade directions, polar plots have a wide range of applications for different types of data. By using radial axes, data can be visualized according to a specific metric or characteristic, while angular positions illustrate the relationship between data points [11].

#### 2.2.2. Absolute Frequency

Port et al. [8] examined the saccades of people from different age groups in the visual search game “Where’s Waldo?” to uncover potential age-related differences in search behavior. Among other aspects, they examined the absolute frequency of saccades by direction, first using histograms in Cartesian coordinate systems (direction on the abscissa, number of saccades on the ordinate) and second using histograms in polar diagrams (radius represents number of saccades, angle represents direction). Both diagrams display the frequency of saccades within defined angular bins.

Samonds et al. [9] investigated the saccadic eye movements of mice in their study and compared them with human saccades to identify cross-species similarities. The mice viewed small and large natural images while their eye movements were measured. Saccade directions were plotted in polar diagrams. To create the impression of a continuous distribution, absolute frequencies across angular bins were connected with a line. However, this procedure is problematic, as it suggests continuity that is not present in the discrete data.

Overall, mapping absolute frequencies of saccades provides a simple visual way to illustrate directional trends, but it comes with important drawbacks. On the one hand, representing frequencies in histograms based on intervals neglects the exact locations of the data [12]. On the other, depicting saccade directions in the Cartesian coordinate system can lead to misunderstandings, particularly because 0° and 360° may be interpreted as different directions [20]. Polar diagrams represent circularity more accurately and make the connection between 0° and 360° explicit (see Figure 4). Nevertheless, they remain bound to discrete binning and may obscure fine-grained variations in the data.

**Figure 5:**
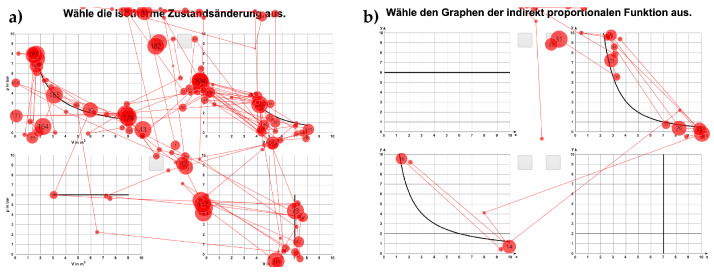
Gaze paths of a learner solving two different tasks on function graphs. (**a**): Original German prompt: “Wähle die isotherme Zustandsänderung aus.” (English translation: “Choose the isothermal change of state.”) (**b**): Original German prompt: “Wähle den Graphen der indirekt proportionalen Funktion aus.” (English translation: “Choose the graph of the indirect proportional function”). Although the total number of saccades differ substantially (169 vs. 29), the relative distribution of vertical (58.6%) and horizontal (41.4%) saccades is identical in both tasks, showing that absolute frequencies can be misleading for comparisons.

The use of absolute frequencies is, however, less suitable for comparing saccade frequencies between different test groups. Figure 5 shows one learner’s eye movements while solving two different tasks [21]. When comparing the absolute frequencies of saccade directions in polar diagrams, differences between the tasks may appear larger than they actually are. An examination of the gaze paths (see Figure 5) highlights how absolute frequency can lead to misinterpretation. When relative frequencies are considered instead, it becomes clear that the ratio of horizontal to vertical saccades is the same in both tasks.

**Figure 6:**
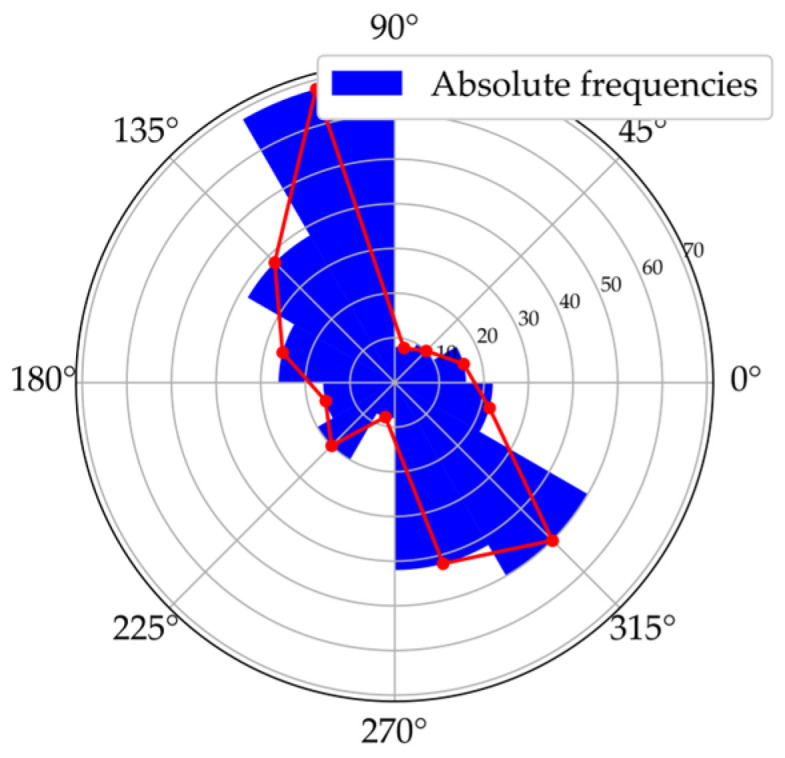
Polar diagram of absolute saccade frequencies. Red dots mark the centers of the individual bars; connecting them with lines can create the visual impression of continuous values between discrete bins.

In addition, inaccuracies can occur when discrete values of absolute frequency within angular bins are connected by a line. This procedure creates the impression of continuity, although the values between the discrete points are in fact unknown, and may suggest a precision that the data do not support [13]. To illustrate this problem in practice, Figure 6 shows an example of how such bin-based visualizations are typically constructed.

While connecting the centers of the bins with lines is a common practice, it formally implies values between the discrete bins that are not known. This does not necessarily lead to misinterpretation, especially when bins are narrow and numerous, in which case the connected line may approximate the underlying distribution. However, when bins are wide or few in number, the resulting line can diverge substantially from the true density and may visually suggest a level of precision that the data do not support. KDE avoids this interpolation by estimating the continuous distribution directly from the data.

#### 2.2.3. Relative Frequency

Klein and Hahn [4] investigated the effects of drawing activities on learning by analyzing learners’ gaze behavior in the context of vector fields. They displayed the relative frequencies of saccade directions in polar diagrams, plotting relative frequency values and connecting them with lines. As with absolute frequencies, the same visual impression of continuity can arise here. Nevertheless, the use of relative frequencies offers a clearer basis for comparing gaze behavior across experimental conditions.

Andersson et al. [10] used a polar bar chart to visually represent the relative frequency of saccades as a function of their direction, thereby accounting for circularity. However, they displayed frequencies of saccade amplitude separately in Cartesian coordinates, disregarding directional information that may be of interest for more detailed analyses.

#### 2.2.4. Probability Distribution

Le Meur and Liu [7] presented a new approach to analyzing eye movements in which they derived a joint probability distribution of saccade amplitudes and saccade directions. This approach makes it possible to consider both the amplitude and the direction of saccades simultaneously and thus gain a more comprehensive understanding of eye movements. They visualized the resulting probability densities continuously as a heatmap in a polar diagram.

Hoyer and Girwidz [3] took a similar approach. However, instead of using a continuous representation in the form of a curve in the polar diagram, they represented the data using discrete circular segments. As a result, the continuity is not immediately apparent.

A key advantage of probability density estimation is that it enables direct comparisons between different conditions or groups, regardless of the absolute number of saccades observed. In addition, it provides a continuous visualization of the distribution [22], which is particularly relevant as saccade directions span a continuous angular range. Kernel density estimates converge more quickly to the true density than histogram-based methods, making them more accurate and efficient at approximating the underlying data distribution, especially when observations are limited [23].

**Figure 7:**
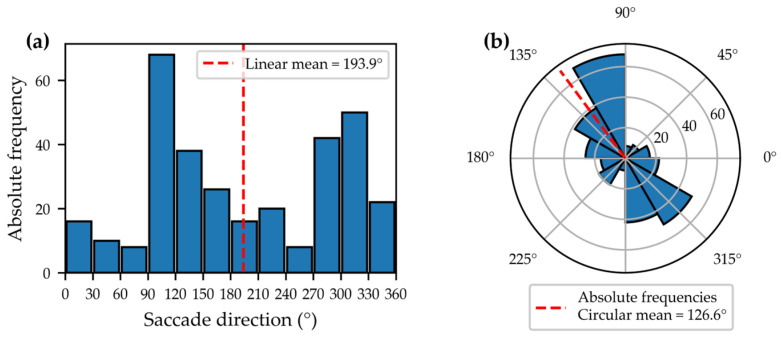
Absolute frequencies of saccade directions as a bar chart in Cartesian coordinates (**a**) and as a polar diagram (**b**). In the Cartesian plot, the arithmetic mean (dashed red line) lies between 150° and 180°, whereas in the polar plot the circular mean is correctly located at around 90°.

However, not every kernel is suitable for every type of data. Saccade directions are circular, meaning that 0° and 360° represent the same direction [20]. This poses a particular challenge for density estimation, as Gaussian kernels are not optimal for capturing circular data. Just as the arithmetic mean is inappropriate for circular variables (see Figure 7), standard kernel density estimation methods for linear data cannot be applied to angular data [13]. This observation highlights the need for a density estimation method that inherently preserves the circular structure of saccade directions, leading to the use of a von Mises kernel.

## 3. Goals of a New Analytical Approach

In this article, we focus on the application of probability density to increase the efficiency and accuracy of saccade analysis. We pursue two main objectives:

1. Our first goal is to precisely visualize the saccade directions by a continuous representation using a suitable probability density in a polar diagram. This approach enables a fine-grained analysis of directional distributions and provides deeper insights into the dynamics of saccade behavior, thereby improving the interpretation of visual perception.
2. Our second goal is to account not only for saccade directions but also for their amplitudes. For this purpose, we apply probability density estimation to both metrics simultaneously, allowing for a comprehensive analysis. By representing both probability densities in one polar diagram, we enable the simultaneous examination of direction and amplitude and thereby promote a holistic analysis of eye movements.

With these methods, we aim to enable an efficient and statistically accurate approach to saccade analysis that can help to link gaze behavior more closely to underlying cognitive processes.

## 4. Saccade Analysis

### 4.1. Kernel Density Estimation for Analyzing the Distribution of Saccade Direction (Goal 1)

To analyze saccade directions, we also apply a density estimation approach. However, in contrast to conventional methods, we use a kernel specifically suited for circular or radial data. The von Mises distribution, often considered the circular analogue of the normal distribution in linear data analysis, provides a statistically robust framework for handling such data [11]. In this distribution, data points are arranged along the circumference of a unit circle, allowing the periodic structure of directional data to be accurately represented [11].

**Figure 8:**
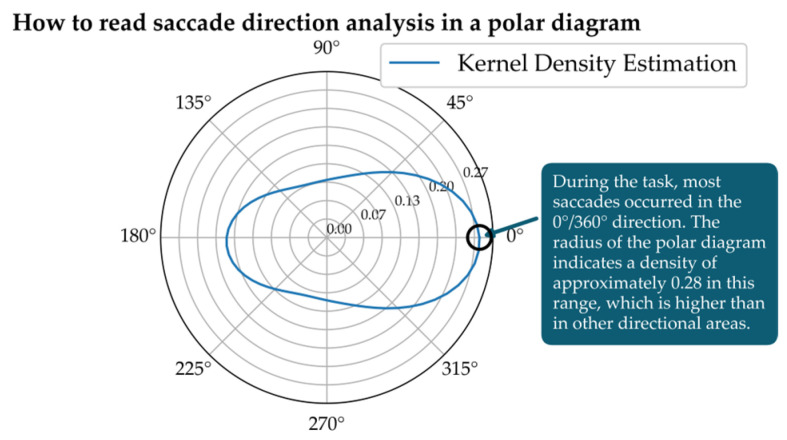
KDE of saccade direction using the von Mises kernel in a polar plot represented as a line graph. Saccade directions near 0°/360° occurred most frequently, where the density is highest. On the visual stimulus, the saccades predominantly pointed forward (regardless of their location on the stimulus).

The estimation is based on combining individual observations around each point on the circle, resulting in a smooth and continuous representation of the underlying distribution (see Figure 8). Figure 8 also explains how to read the information displayed. The Python script (version 3.10) used to generate this visualization is provided as Python Code S1 in the Supplementary Materials.

Figure 8 shows the resulting KDE as a line plot on the polar diagram. Density values are plotted on the radial axis. A greater distance from the center indicates higher densities. The angles can be read around the circle. Figure 8 shows that saccades occur most frequently in the gaze directions from 330 to 30 degrees. This type of analysis has been used, for example, when students have interpreted graphs of indirect proportional functions. By analyzing the direction of the saccades, it became clear how the students viewed the graph.

**Figure 9:**
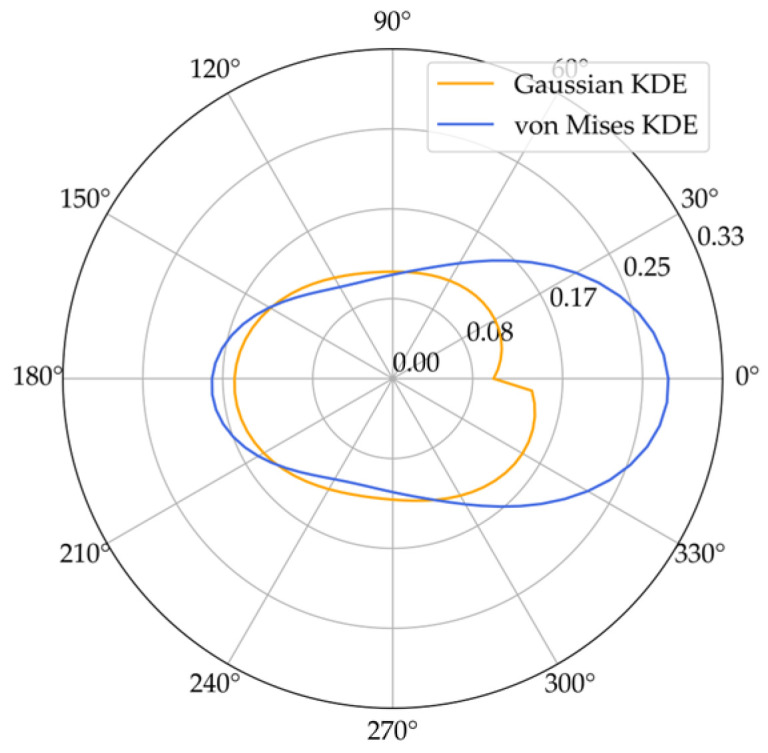
Kernel density estimation of saccade direction using the Gaussian kernel (orange line) and the von Mises kernel (blue line).

To illustrate the difference between the Gaussian kernel and the von Mises kernel, Figure 9 shows both kernel density estimates of the same dataset. The blue line represents the distribution estimates using a von Mises kernel, while the orange line represents the distribution estimates using a Gaussian kernel. A key difference between these two estimates lies in their adaptation to circular data. This is evident in the range between 330 and 30 degrees. In this section, the specific characteristics of the saccade direction density are not discernible. The von Mises kernel allows for a more consistent and precise adaptation to circular data. Therefore, the von Mises kernel provides a smoother and more accurate density estimation compared to the Gaussian kernel density estimate, offering a better fit to the original data (see Figure 9).

### 4.2. Kernel Density Estimation for Analyzing the Distribution of Saccade Direction and Saccade Amplitude (Goal 2)

**Figure 10:**
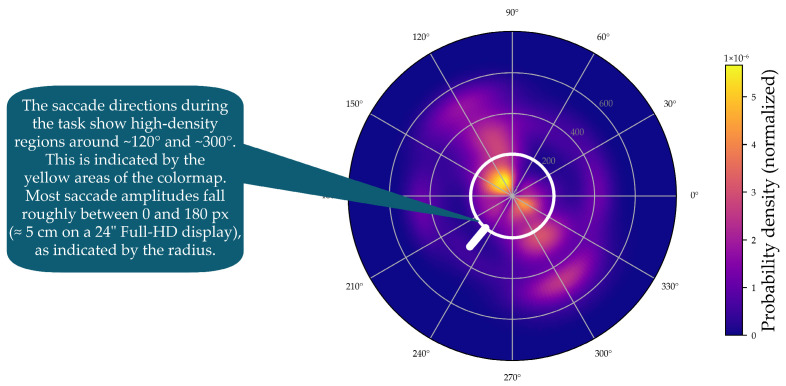
Two-dimensional KDE of saccade direction and amplitude in polar coordinates. Angle indicates saccade direction, radius indicates saccade amplitude and color represents density.

To visualize the kernel density estimates for saccade direction and saccade amplitude simultaneously, displaying them as a line graph in a polar plot is not sufficient. A heat map is needed to show the density of both saccade direction and saccade amplitude (see Figure 10).

In these polar representations, saccade direction is encoded by the angular coordinate, while saccade amplitude is encoded by the radial coordinate. Saccade amplitudes were provided in degrees of visual angle by the Tobii Pro Lab software (version 24.21) and, for visualization in polar coordinates, were converted to screen pixels using the horizontal pixels-per-degree factor (39.04 px/deg), derived from the monitor’s physical dimensions, screen resolution, and viewing distance [24].

Figure 10 also explains how to read the information displayed. The script provides as Python Code S2 in the Supplementary Materials is used to generate such kernel density estimates in a polar plot.

**Figure 11:**
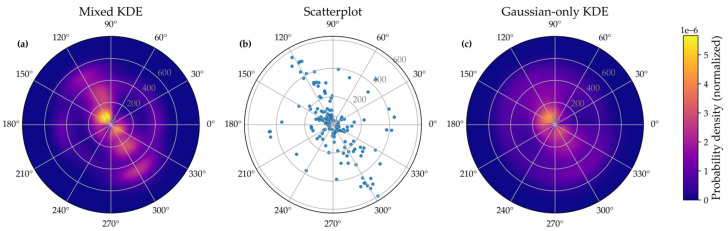
Two-dimensional KDE of saccade direction and amplitude in polar coordinates. Angle indicates saccade direction, radius indicates saccade amplitude and color represents density. (**a**) Mixed KDE using the von Mises kernel for direction and the Gaussian kernel for amplitude. (**b**) Raw data points plotted by direction and amplitude. (**c**) Gaussian-only KDE using Gaussian kernels for both direction and amplitude.

Figure 11a shows the polar plot with the probability densities of saccade directions and saccade amplitudes, created using mixed kernel density estimation. The color scale represents the mixed density. The mixed kernel density estimate represents a normalized joint probability density over direction and amplitude. Densities are normalized using the polar area element such that they integrate to one; consequently, color-coded density values reflect relative likelihoods rather than absolute frequencies and are independent of the number of participants. Warmer colors indicate higher density values. Saccade amplitudes are plotted along the radial axis, while saccade directions are represented as angular positions. Directions between 90 and 150 degrees and 270 and 330 degrees are particularly pronounced. Most saccades have amplitudes below 200 pixels, but some longer saccades are visible in lighter purple tones.

As a baseline, we used a Gaussian kernel density estimator in the same polar coordinate system, applying Gaussian kernels to both saccade direction and amplitude. This “Gaussian-only” KDE provides a direct comparison to the mixed KDE because both methods operate on the same (*θ*, *r*) space and share the same product structure, differing only in the choice of kernel for the directional component.

Figure 11c shows the resulting density map. Gaussian kernels treat direction as a linear variable. As a consequence, directional patterns can become less distinct. This effect is especially visible near the 0°/360° boundary. Figure 11b shows the raw saccades for comparison.

When comparing Figure 11a,c, it becomes clear that the mixed KDE preserves directional structure more effectively. The mixed KDE shows consistent density around the circular boundary and captures narrow directional clusters. The Gaussian-only KDE smooths these features more strongly and therefore loses some of this structure.

To quantify the difference between the Gaussian-only KDE and the mixed von Mises–Gaussian KDE, we computed the Jensen–Shannon divergence (JSD) between the two density estimates. Both KDEs were evaluated on the same polar grid and normalized using a polar area element to obtain comparable probability distributions. The normalized JSD was 0.19, meaning that the Gaussian-only estimate differed by about 19% of the maximal possible divergence between two distributions. Although the absolute difference is modest, it is systematic and reflects the fact that Gaussian kernels do not account for the circular nature of directional data, which the von Mises kernel captures more faithfully. Here, the Jensen–Shannon divergence serves to complement the visual comparison by quantitatively illustrating methodological differences between the two density approaches, rather than providing a validation against a known reference distribution.

### 4.3. Saccades and Cognitive Processing

Cognitive processing during task engagement unfolds through the learner’s continuous interaction with the presented stimulus. Traditional pre- and post-tests can only capture performance before and after this interaction, but they do not provide access to the processes that occur while the learner is working with the material. Eye tracking, in contrast, offers objective, time-resolved process data that directly reflect how users interact with a stimulus during task execution and how information processing evolves over time [25]. Saccadic eye movements indicate moment-to-moment decisions about where visual attention is allocated and how information is traversed.

Within this interaction, different saccade parameters capture distinct functional aspects of cognitive processing. Saccade direction reflects the orientation and pathways through which information is read or compared, such as horizontal or vertical traversal of a diagram or text. Saccade amplitude, in turn, reflects the degree of attentional reorganization between information units and has been shown to be sensitive to task demands and processing strategies rather than purely spatial constraints [19]. Short saccades are typically associated with local, goal-directed processing, whereas longer saccades indicate exploratory behavior and increased organizational demands.

Importantly, cognitive strategies are not expressed in isolated eye-movement parameters but in the systematic coupling of multiple parameters during interaction with the stimulus. Analyzing saccade direction and amplitude jointly therefore allows inferences about how reading orientation and organizational effort are coordinated in real time. The joint distribution captures whether specific traversal patterns are associated with efficient, structured processing or with exploratory, less organized strategies—insights that cannot be derived from separate analyses of direction and amplitude alone. The mixed KDE highlights structured direction–amplitude relations that are consistent with goal-directed processing, whereas the Gaussian-only KDE smooths these relations and renders them less distinct (see Figure 11).

## 5. Application Example: Selection Processes in Task Settings

Saccadic eye movements play a crucial role in selection and decision-making, for example when solving multiple-choice tasks in everyday contexts such as university exams or driving tests. To demonstrate the added value of our 2D mixed KDE approach, we apply it to a subset of eye-tracking data from a previously published study [21]. The original study investigated learners’ gaze behavior while distinguishing between two thermodynamic processes (isothermal vs. adiabatic).

Since the present article focuses exclusively on the methodological contribution, we provide only the technical details required to understand and replicate the 2D mixed KDE analysis.

### 5.1. Data Collection and Preprocessing

**Figure 12:**
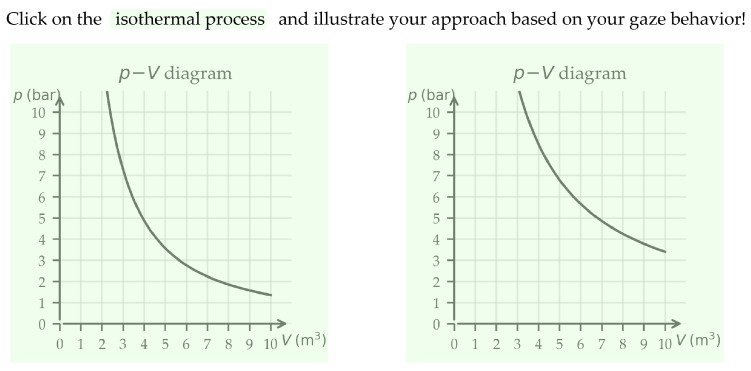
Task design with defined Areas of Interest (AOIs) for the analysis of saccade directions and saccade amplitudes. The AOIs cover the key term (240 × 30 px) in the question and the two answer diagrams (each 450 × 450 px), enabling targeted analysis of gaze shifts during the selection process.

Eye movements were recorded using a Tobii Pro Fusion eye tracker operating at 120 Hz. Saccades were detected using the Velocity-Threshold Identification (I-VT) algorithm implemented in the Tobii Pro Lab software, with an average gaze accuracy of approximately 0.40°. The eye tracker was mounted below a 24-inch monitor (1920 × 1080 pixels) on which 80 participants completed a graph-identification task. The present analysis follows a stimulus-based approach, in which saccade directions and amplitudes are interpreted relative to the spatial structure of the task layout. To this end, Areas of Interest (AOIs) were defined directly on the stimulus and served as the spatial reference frame for all subsequent analyses. Specifically, three AOIs were specified: (1) the key term in the task prompt (240 × 30 pixels) and (2–3) the two graphical answer options (each 450 × 450 pixels; see Figure 12). Only saccades occurring within or between these predefined AOIs were included in the analysis. This restriction ensures that the analyzed eye movements reflect gaze shifts that are functionally relevant to the selection process between the linguistic cue and the graphical representations, rather than peripheral or unrelated eye movements. Saccade direction was extracted from Tobii Pro Lab as an absolute angle in degrees, defined with respect to the horizontal screen axis (0–360°). Saccade amplitude was provided by the software in degrees of visual angle, representing the angular distance between two successive fixations, and was converted to pixels for visualization as described in Section 4.2. Additional methodological details of the original study are reported in [21].

### 5.2. Grouping

To illustrate how the mixed KDE can reveal differences in direction–amplitude patterns, we contrast two groups of participants based on task performance: correct responders (n_1_ = 42) and incorrect responders (n_2_ = 38). This grouping is used exclusively as an example to demonstrate how 2D mixed KDE-based visualization can capture differences in gaze behavior across participant subgroups.

### 5.3. Comparison of the Application of 2D Mixed KDE and Gaussian-Only KDE to Real Data

**Figure 13:**
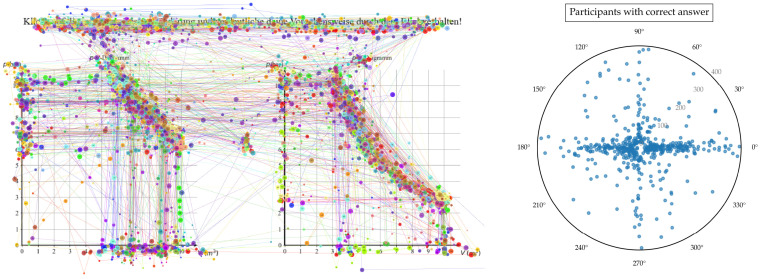
Saccadic eye movements of all participants who solved the task correctly.

**Figure 14:**
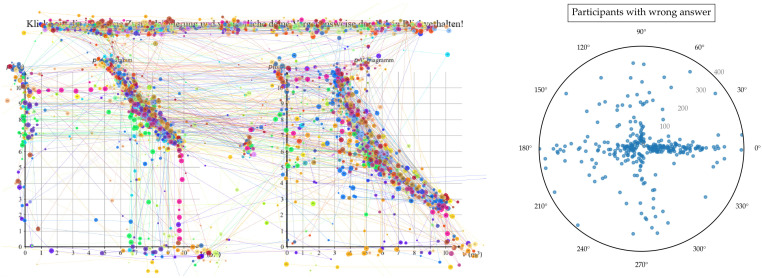
Saccadic eye movements of all participants who failed to solve the task.

Figure 13 and Figure 14 display the aggregated gaze paths of participants who solved the task correctly (Figure 13) and incorrectly (Figure 14). To solve the task, learners needed to select pairs of values and check for product equality, which required both vertical and horizontal saccades. As shown in Figure 13, these saccades are clearly visible among participants who solved the task correctly. Interestingly, horizontal and vertical saccades also appear among participants who solved the task incorrectly (Figure 14). However, it is difficult to determine from visual inspection alone whether these occurred to a greater or lesser extent, underscoring the need for quantitative analysis.

The raw data underlying the group comparison are shown in Figure 13 and Figure 14 using two conventional visualization approaches: aggregated gaze paths and scatterplots of individual saccades. These representations allow inspection of overall viewing behavior and unsmoothed saccade distributions but do not readily reveal systematic differences in the joint distribution of saccade direction and amplitude between groups.

**Figure 15:**
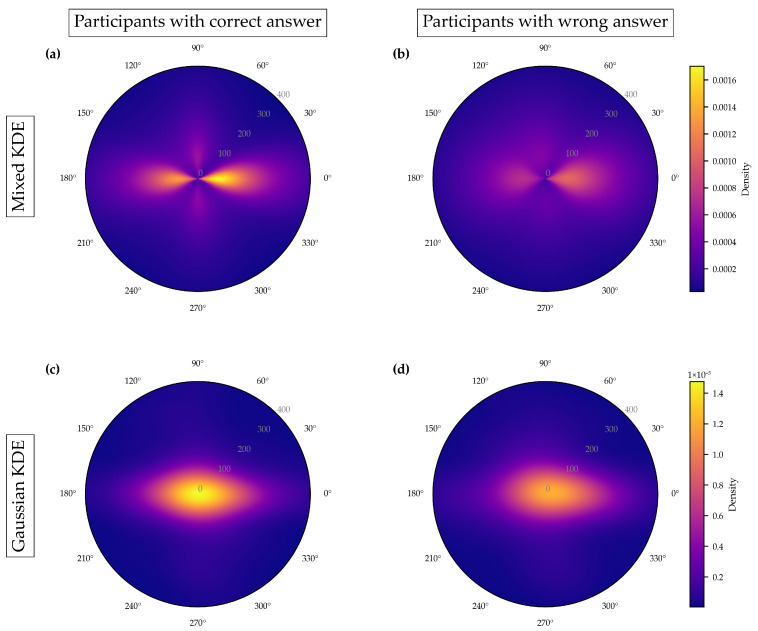
Mixed KDE (**a**,**b**) and Gaussian KDE (**c**,**d**) of saccade direction and amplitude. (**a**,**c**): Participants with correct answers; (**b**,**d**): Participants with incorrect answers. In contrast to the raw gaze paths (Figure 13 and Figure 14), the mixed KDE reveals clear differences in gaze strategies between the two groups.

Figure 15 builds on these raw-data representations by applying the mixed kernel density estimation to the same underlying saccade data. By aggregating individual saccades into a normalized joint density over direction and amplitude, the KDE reveals stable group-level patterns that are difficult to extract from the unsmoothed visualizations. Importantly, the density structures observed in Figure 15 are already hinted at in the raw data shown in Figure 13 and Figure 14, indicating that the KDE does not introduce artificial patterns but rather provides a structured summary of existing tendencies.

The mixed KDE shown in Figure 15 reveals clear differences between participants with correct and incorrect answers. For correct answers, saccades occurred predominantly in the horizontal direction and followed a narrow structure. This pattern is consistent with a more targeted and systematic visual strategy, characterized mainly by shorter to medium-amplitude saccades. In contrast, incorrect answers were associated with more diffuse patterns, reduced directional consistency, and longer saccades. This pattern is consistent with more exploratory and less structured search behavior, which has been associated in previous work with increased cognitive uncertainty. Gaussian KDE is suitable for visualizing general amplitude distributions. However, it has limitations in handling circular data: it blurs directional differences due to isotropic smoothing, and is highly dependent on bandwidth selection.

As shown in Figure 15, participants who answered incorrectly exhibited longer and less directed saccades, reflecting unsystematic search behavior and increased cognitive uncertainty. Rather than following a clear strategy, their gaze patterns indicated inefficient problem solving. These group differences are presented solely to illustrate how the proposed method can be applied and interpreted; they are not intended as empirical claims about strategy effectiveness beyond this exemplary dataset.

The observed differences thus reflect not only outcome-related group differences, but differences in how participants interacted with the visual structure of the task during processing. In line with the process-oriented perspective outlined above, the joint direction–amplitude patterns capture how visual attention was organized in real time while engaging with the stimulus.

It is important to note that the mixed KDE does not aim to classify individual learners or predict correctness on a trial-by-trial basis. Instead, it provides a distribution-based characterization of gaze behavior at the group level. Figure 15 therefore should not be interpreted as a diagnostic tool for individual performance, but as a method for revealing systematic tendencies in how visual information is processed under different outcome conditions. This highlights the role of the mixed KDE as a process-oriented analytical tool rather than a purely descriptive visualization.

Our method thus enables a psychologically meaningful interpretation of the selection process: inefficient strategies can be identified from characteristic saccade patterns, which in turn could be used in digital learning environments to provide adaptive support when learners fail to use effective strategies.

The potential of this approach extends beyond multiple-choice tasks. Such tasks are particularly suitable for analysis, as their visual layout is highly standardized (e.g., identical spacing and positioning of options). This uniformity ensures that subtle differences in gaze behavior are not confounded by differences in visual design and can therefore be detected more reliably.

In physics education, for instance, interpreting pressure–volume (*p*-*V*) diagrams requires integrating information from different regions of a graph. In a study by [21], learners displayed distinct strategies: some focused on the function graph, producing predominantly diagonal saccades while others relied on individual data points, reflected in horizontal and vertical saccades. Our 2D mixed KDE approach made these patterns visible (and interview data provided further support).

## 6. Summary

In the following, we consider an exemplar eye-tracking dataset (Dataset S1, see Supplementary Materials) to summarize and compare different approaches for representing and evaluating saccade directions and amplitudes.

**Figure 16:**
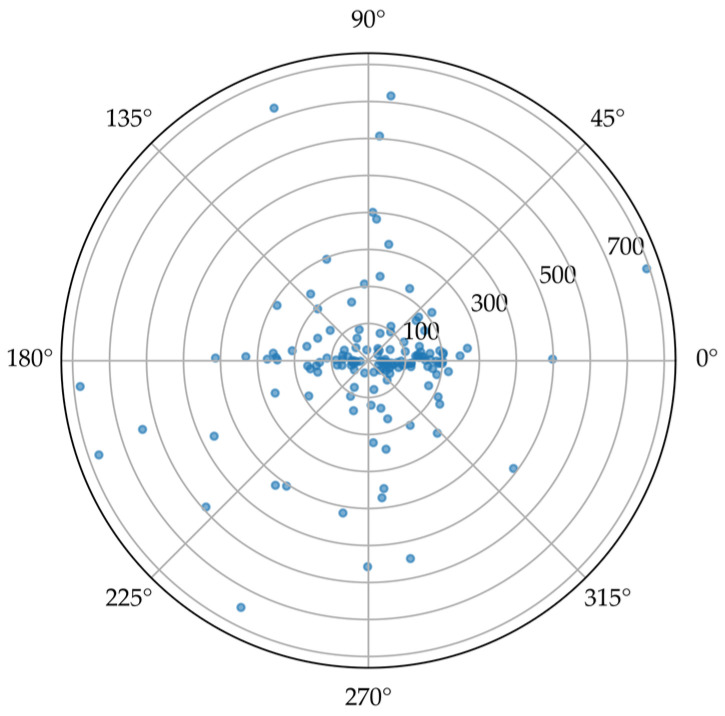
Raw saccade data plotted in polar coordinates. Each point represents a single saccade, with direction shown as angle and amplitude shown as radial distance from the center. Larger amplitudes appear farther from the center of the plot.

The corresponding raw saccade directions and amplitudes are shown in Figure 16.

Based on this dataset, Table 1 summarizes established and newly developed formats for representing and evaluating saccade directions and amplitudes, including their advantages and disadvantages, and associated cognitive processes.

Importantly, the fewer disadvantages a method entails, the more precise and valid the resulting statements about cognitive processes become.

## 7. Discussion and Outlook

The method presented in this paper applies KDE with a von Mises kernel to the analysis of saccade directions and a mixed 2D KDE for the combined analysis of saccade directions and amplitude. It advances previous approaches by accounting for the circular nature of gaze direction data and providing a more precise estimation of their distribution. Earlier studies—such as that of Le Meur and Liu [7]—used Gaussian kernels, which are suboptimal for circular data like saccade directions. In contrast, the von Mises kernel preserves cyclic similarity, ensuring that 0° and 359° are modeled as neighboring values.

A key advantage of this method is the computation of continuous probability distributions. Unlike discrete frequency histograms, KDE enables finer differentiation of gaze directions and allows for the derivation of psychologically interpretable metrics such as skewness, dominance of directional ranges, or multimodal patterns. These characteristics enable nuanced analyses of visual strategies at both individual and group levels, as well as across different task conditions. The joint representation of saccade direction and amplitude provides new opportunities to investigate cognitive processes, particularly in visually complex tasks such as interpreting function graphs or solving multiple-choice tasks. It allows researchers to determine, for instance, whether attention is directed more toward structural elements (e.g., axis labels) or content-related information (e.g., data trends). In this way, the method contributes to the empirical grounding of theoretical models of visual cognition.

A further promising application is linking KDE-derived parameters to performance outcomes such as accuracy, response time, or error rates. This shifts the method from a purely descriptive tool toward explanatory use. In our example, longer and less structured saccades were associated with lower response rates—reflecting inefficient visual strategies.

As with all kernel-based methods, the resulting density estimates depend on parameter choices such as angular resolution, bandwidth, and concentration estimates. Alternative reasonable parameter settings may lead to modest variations in local density structure, while the overall directional and amplitude-related patterns remain stable.

Despite these strengths, limitations remain. The current approach does not yet incorporate saccade velocity, which may also provide insights into cognitive processing. Moreover, similar to the Gaussian kernel in linear data, the von Mises kernel assumes unimodality. In cases where gaze patterns are strongly multimodal, the density estimation may excessively smooth the data, thereby obscuring relevant structure.

## Figures and Tables

**Table 1:** Representation and evaluation methods of saccade directions and amplitudes, including their advantages and disadvantages.

Saccade Metric	Visualization	Icon	Cognition	Disadvantages	Advantages
Absolute frequencies					
Saccade direction	Cartesian histogram	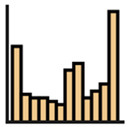	Reading process	No continuity; interval-based representation; no group comparisons	—
Saccade direction	Polar histogram	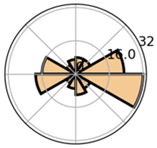	Reading process	No group comparisons; interval-based representation	Improved continuity (circular layout);
Relative frequencies					
Saccade direction	Polar histogram	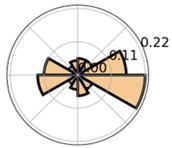	Reading process	interval-based representation	Improved continuity (circular layout); group comparisons possible
Saccade direction	Polar histogram with line graph	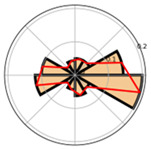	Reading process	No estimation methods, imprecise directional trends	Improved continuity (circular layout); group comparisons possible; continuous line graph
Saccade direction and amplitude	Polar histogram and Cartesian histogram	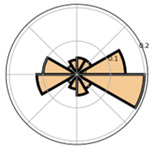 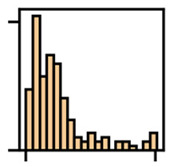	Reading and organizational processes	Separate metrics, loss of integrated information	—
Gaussian-only KDE					
Saccade direction	Polar diagram with line graph	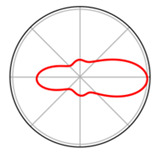	Reading process	Risk of misestimating directionality	Continuity preserved; direction not interval-based
Saccade direction and amplitude	Polar diagram with heatmap	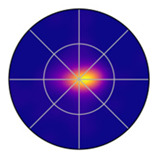	Reading and organizational processes	Risk of misestimating directionality	Integrated representation of both metrics
Saccade direction and amplitude	Polar diagram with discrete segments	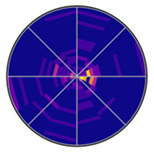	Reading and organizational processes	Risk of misestimating directionality; Segmentation limits continuity	Integrated representation of both metrics
von Mises KDE					
Saccade direction	Polar diagram with line graph	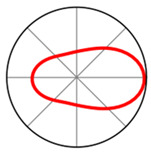	Reading process	—	Continuity of data preserved
Saccade direction and amplitude	Polar diagram with heatmap	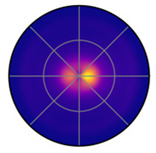	Reading and organizational processes	—	Integrated representation of both metrics

All visualization and evaluation methods were applied to the same exemplar dataset; the raw saccade directions are displayed in Figure 16 (polar scatterplot).

## Data Availability

The datasets analyzed during the current study are available in the Supplemental Materials. Additional data can be requested from the corresponding author.

## Supplementary Materials

The following supporting information can be downloaded at: https://www.mdpi.com/article/10.3390/jemr19010010/s1, Python Code S1 (kernel density estimation of saccade direction in a polar plot represented as a line graph), Python Code S2 (two-dimensional kernel density estimation of saccade direction and saccade amplitude displayed as a colored mesh), Dataset S1, and Dataset S2. Refs. [24,26] has been cited in Supplementary Materials file.
