# Supplementary material for: Eye Movement Analysis: A Kernel Density Estimation Approach for Saccade Direction and Amplitude
Source: J Eye Mov Res. 2026 Jan 19;19(1):10. doi: 10.3390/jemr19010010 (PMC12922154; doi:10.3390/jemr19010010)
Supplement: Supplementary file 1 [file jemr-19-00010-s001.zip › Python Code S1.pdf]

Journal of
Eye Movement Research

MDPI

Supplementary Materials: Python Code S1

Kernel Density Estimation of Saccade Direction in a Polar Plot Represented as a Line Graph

```
import pandas as pd                  # Pandas for reading and processing tabular data
import numpy as np                   # NumPy for numerical calculations
import matplotlib.pyplot as plt      # Matplotlib for plotting
import math                          # math for mathematical functions
from scipy.stats import vonmises     # von Mises distribution from SciPy for KDE

# ==========================
# 1) Load data
# ==========================
# Read only the saccade direction column from the Excel file.
# Missing values encoded as 'null' are treated as NaN.
xlsx_file = 'path_to_your_file.xlsx'
df = pd.read_excel(xlsx_file, na_values='null', usecols=['Saccade_direction'])

# Convert degrees to radians (assuming the input is in 0…360 degrees)
df['Saccade_direction'] = np.radians(df['Saccade_direction'])

# ==========================
# 2) Matplotlib styling
# ==========================
# Set a consistent typographic style.
plt.rcParams['font.family'] = 'Palatino Linotype'
plt.rcParams['font.size'] = 9
plt.rcParams['axes.labelsize'] = 9
plt.rcParams['axes.titlesize'] = 9
plt.rcParams['xtick.labelsize'] = 7
plt.rcParams['ytick.labelsize'] = 7

# ==========================
# 3) Von Mises KDE for circular data
# ==========================
# Estimate the concentration parameter kappa from the mean resultant length R.
# This follows the approximation proposed by Zulkipli et al. [26].
# Each saccade direction is interpreted as a unit vector on the unit circle:
# Computing the mean cosine and sine corresponds to averaging these vectors
# component-wise. The resulting mean resultant vector summarizes the dominant
# direction and the directional consistency of the data.
# The length of the mean resultant vector (R) serves as a measure of directional
# concentration and is used to estimate the concentration parameter (kappa)
# of the von Mises distribution.
# Note: This is not a simple arithmetic mean of angles, but a vector-based
# averaging approach required for circular (angular) data.
c = np.mean(np.cos(df['Saccade_direction']))
```

```
s = np.mean(np.sin(df['Saccade_direction']))
R = math.sqrt(c**2 + s**2)
# ========================
# Estimation of the concentration parameter kappa
# ========================
# The concentration parameter kappa of the von Mises distribution is estimated
# from the mean resultant length R. Since the relationship between R and kappa
# has no closed-form inverse, we apply a piecewise approximation following
# Zulkipli et al. [26], which is commonly used in circular statistics.
# Small R values indicate weak directional concentration (low kappa),
# whereas larger R values correspond to stronger concentration (high kappa).
if R < 0.53:
    kappa = 1 / (2 * R + R**3 + (5 * R**5) / 6)
elif R < 0.85:
    kappa = 1 / (1.39 * R + 0.43 / (1 - R) - 0.4)
else:
    kappa = 1 / (3 * R - 4 * R**2 + R**3)

# Evaluate the KDE on an evenly spaced angular grid.
# num_bins_kde controls the angular resolution of the resulting curve.
# Angular resolution of the evaluation grid for the KDE.
# num_bins_kde = 72 corresponds to 5° steps (360° / 72).
# This defines only the sampling resolution of the estimated density curve
# and does not imply binning or discretization of the underlying saccade data.
# Increasing this value yields a visually smoother curve without altering
# the estimated density structure.
num_bins_kde = 72
angle_kde = np.linspace(0, 2 * np.pi, num_bins_kde, endpoint=False)

# ========================
# Circular kernel density estimation
# ========================
# For each angle on the evaluation grid, a von Mises kernel is centered at
# that angle and evaluated for all observed saccade directions.
# The kernel values are summed and normalized by the sample size, yielding
# the estimated directional density at that angle.
density_values = []
for angle in angle_kde:
    kernel_value = vonmises.pdf(df['Saccade_direction'] - angle, kappa)
    normalized_density = np.sum(kernel_value) / len(df)
    density_values.append(normalized_density)

# Close the density curve to ensure circular continuity.
# The first density value (at 0°) is appended at the end (360°),
# so that the polar plot forms a closed curve without a visual gap.
density_values.append(density_values[0])
angle_kde = np.append(angle_kde, angle_kde[0])

# ========================
# 4) Polar plot (for manuscript figure)
# ========================
```

```
# Create a polar coordinate system to visualize directional data.
# Polar plots preserve the circular nature of angles and allow direct
# interpretation of direction (angle) and density (radius).
fig, ax_polar = plt.subplots(
    figsize=(6, 6),
    subplot_kw={'projection': 'polar'}
)
# Plot the circular kernel density estimate as a continuous curve.
# Angle corresponds to saccade direction, radius represents estimated density.
ax_polar.plot(angle_kde, density_values, label='Kernel Density Estimation')
# Set a descriptive title
ax_polar.set_title("Kernel Density Estimation of Saccade Directions",
fontweight="bold"
)

# --- Fix the outer radius limit (as requested) ---
# This ensures consistent scaling across figures / groups.
r_max = 0.3
ax_polar.set_ylim(0, r_max)
ax_polar.set_autoscale_on(False)

# Radial ticks: label only every second tick to reduce visual clutter.
r_ticks = np.linspace(0, r_max, num=10)
r_tick_labels = [f"{tick:.2f}" if i % 2 == 0 else "" for i, tick in enumerate(r_ticks)]
ax_polar.set_yticks(r_ticks)
ax_polar.set_yticklabels(r_tick_labels)

# Angular ticks (0° to 315° in 45° steps).
ax_polar.set_xticks(np.linspace(0, 2*np.pi, 8, endpoint=False))
ax_polar.set_xticklabels(
    [f'{int(np.degrees(a))}°' for a in np.linspace(0, 2*np.pi, 8, endpoint=False)]
)

ax_polar.legend(loc="upper right")

# 6) Save figure
# =========================
# Save at 600 dpi with tight bounding box.
plt.tight_layout()
plt.savefig("yout_path/fig.png", dpi=600, bbox_inches="tight")
plt.show()
```
