# Supplementary material for: Eye Movement Analysis: A Kernel Density Estimation Approach for Saccade Direction and Amplitude
Source: J Eye Mov Res. 2026 Jan 19;19(1):10. doi: 10.3390/jemr19010010 (PMC12922154; doi:10.3390/jemr19010010)
Supplement: Supplementary file 1 [file jemr-19-00010-s001.zip › Python Code S2.pdf]

Journal of
Eye Movement Research

MDPI

## Supplementary Materials. Python Code S2

## Two-Dimensional Kernel Density Estimation of Saccade Direction and Saccade Amplitude Displayed as Colored Mesh

```
import pandas as pd               # Pandas for reading and processing tabular data
import numpy as np                # NumPy for numerical calculations
import matplotlib.pyplot as plt   # Matplotlib for plotting
import math                       # math for mathematical functions
#von Mises and Gaussian distribution from SciPy for KDE
from scipy.stats import gaussian_kde, vonmises

# ========================
# 1) Load data
# ========================
# Read saccade amplitude and direction from Excel file
# Missing values encoded as 'null' are treated as NaN.
xlsx_file = 'path_to_your_file.xlsx'
df = pd.read_excel(xlsx_file, na_values='null',
usecols=['Saccade_amplitude', 'Saccade_direction']
).dropna()   # Remove incomplete saccade records (NaN values)

# Convert saccade directions from degrees to radians (circular variable)
# Modulo operation ensures values lie within [0°, 360°)
theta_data = np.radians(df["Saccade_direction"].astype(float) % 360)

# Convert saccade amplitude from degrees of visual angle to screen pixels
# using a monitor-specific pixels-per-degree factor (39.04 px/deg)
#In these polar representations, saccade direction is encoded by the angular
#coordinate, while saccade amplitude is encoded by the radial coordinate.
#Saccade amplitudes were provided in degrees of visual angle by the Tobii Pro Lab
#software and were converted to screen pixels for visualization purposes. This
#conversion was performed using a horizontal pixels-per-degree factor
#(39.04 px/deg), derived from the monitor's physical dimensions, screen resolution,
# and viewing distance [24].
radius_data = df["Saccade_amplitude"].astype(float) * 39.04

# ========================
# 2) Matplotlib style settings
# ========================
plt.rcParams['font.family'] = 'Palatino Linotype'
plt.rcParams['font.size'] = 9
plt.rcParams['axes.labelsize'] = 9
plt.rcParams['axes.titlesize'] = 9
plt.rcParams['xtick.labelsize'] = 7
plt.rcParams['ytick.labelsize'] = 7

# ========================
```

```
# 3) Evaluation grid
# ========================
# Angular resolution of the evaluation grid (2° steps)
theta_points = np.linspace(0, 2*np.pi, 180)

# Radial grid extending slightly beyond maximum observed amplitude
max_radius = float(radius_data.max()) + 200
radius_points = np.linspace(0, max_radius, 80)[::-1]    # reversed for polar plotting

# ========================
# 4) Estimate concentration parameter kappa
#       (Zulkipli approximation)
# ========================
# Estimate the concentration parameter kappa from the mean resultant length R.
# This follows the approximation proposed by Zulkipli et al. [26].
# Each saccade direction is interpreted as a unit vector on the unit circle:
# Computing the mean cosine and sine corresponds to averaging these vectors
# component-wise. The resulting mean resultant vector summarizes the dominant
# direction and the directional consistency of the data.
# The length of the mean resultant vector (R) serves as a measure of directional
# concentration and is used to estimate the concentration parameter (kappa)
# of the von Mises distribution.
# Note: This is not a simple arithmetic mean of angles, but a vector-based
# averaging approach required for circular (angular) data.
num_sac = len(theta_data)
c = np.sum(np.cos(theta_data)) / num_sac
s = np.sum(np.sin(theta_data)) / num_sac
R = math.sqrt(c**2 + s**2)

# The concentration parameter kappa of the von Mises distribution is estimated
# from the mean resultant length R. Since the relationship between R and kappa
# has no closed-form inverse, we apply a piecewise approximation following
# Zulkipli et al. [26], which is commonly used in circular statistics.
# Small R values indicate weak directional concentration (low kappa),
# whereas larger R values correspond to stronger concentration (high kappa).
if R < 0.53:
    kappa = 1 / (2 * R + R**3 + 5 * R**5 / 6)
elif R < 0.85:
    kappa = 1 / (1.39 * R + 0.43 / (1 - R) - 0.4)
else:
    kappa = 1 / (3 * R - 4 * R**2 + R**3)

# ========================
# 5) Mixed density estimation
#       (von Mises × Gaussian KDE)
# ========================
# Computes a mixed kernel density estimate for circular (angular) and linear
# variables.
#Angular distributions are modeled using a von Mises distribution,
#while amplitudes are modeled using a Gaussian KDE. The joint density
#is obtained by multiplying both components and averaging across
```

```
#all saccades.
def mixed_kde(theta_data, radius_data, theta_points, radius_points, kappa):
        density = np.zeros((len(radius_points), len(theta_points)))
        linear_kde = gaussian_kde(radius_data)

    for t in theta_data:
        circular_density = vonmises.pdf(theta_points, kappa, loc=t)
        linear_density = linear_kde(radius_points)
        density += np.outer(linear_density, circular_density)

    return density / len(theta_data)

density = mixed_kde(theta_data, radius_data, theta_points, radius_points, kappa)
# ========================
# 6) Polar density plot
# ========================
fig = plt.figure(figsize=(8, 5), dpi=600)

# Polar axis placed to the right to allow space for explanatory callout
ax = fig.add_axes([0.38, 0.12, 0.50, 0.78], projection="polar")

T, Rm = np.meshgrid(theta_points, radius_points)
pcm = ax.pcolormesh(T, Rm, density, cmap="plasma", shading="auto")

# Colorbar representing density magnitude
cax = fig.add_axes([0.95, 0.18, 0.015, 0.64])
fig.colorbar(pcm, cax=cax)

# Centered bold title for the entire figure
fig.suptitle(
"Mixed KDE", fontsize=12, fontweight="bold", ha="center"
)
plt.tight_layout(rect=[0, 0, 1, 0.95])

# Radial axis formatting
radial_ticks = np.arange(0, max_radius + 1, 200)
ax.set_rticks(radial_ticks)
ax.set_rlabel_position(45)
ax.set_yticklabels([f"{t:.0f}" for t in radial_ticks], color="grey")

# Angular axis formatting
ax.set_xticks(np.linspace(0, 2*np.pi, 12, endpoint=False))
ax.set_xticklabels([f"{d}°" for d in range(0, 360, 30)])

# ========================
# 7) Save figure
# ========================
plt.tight_layout(rect=[0, 0, 1, 0.95])
plt.savefig("/path/fig_appendix_b.png", dpi=600, bbox_inches="tight")
plt.show()
```
